# Statins inhibit proliferation and cytotoxicity of a human leukemic natural killer cell line

**DOI:** 10.1186/2050-7771-1-33

**Published:** 2013-12-20

**Authors:** Jon Crosbie, Marc Magnussen, Ryan Dornbier, Alexandra Iannone, Timothy A Steele

**Affiliations:** 1 Department of Microbiology and Immunology, Des Moines University, 3200 Grand Ave., 50312 Des Moines, Iowa, USA; 2 St. Louis University, 221 North Grand Blvd., 63103 St., Louis, Missouri, USA

**Keywords:** Aggressive natural killer cell leukemia, Statins, Proliferation, Cytotoxicity

## Abstract

**Background:**

Natural killer cells comprise the body’s first line of defense against virus-infected cells. As is true of all lymphocytes, natural killer cell malignancies can develop, however natural killer cell leukemias can be very difficult to treat due to their intrinsic resistance to chemotherapeutic agents. With the recent understanding that statin drugs may have anti-cancer properties, our investigations have focused on the ability of statins to inhibit the growth and cytotoxicity of the YT-INDY natural killer cell leukemia cell line.

**Results:**

Our findings indicate that several statin compounds can inhibit YT-INDY proliferation disrupt cell cycle progression and abrogate natural killer cell cytotoxicity. Since natural killer cell leukemia cytotoxicity may play a role in the pulmonary damage seen in these patients, this is an important finding. Cytotoxicity, proliferation and cell cycle progression could be restored by the addition of mevalonate, signifying that the statin effects are brought about through HMG CoA reductase inhibition. The mevalonate pathway intermediate geranylgeranyl pyrophosphate, but not other intermediates in the mevalonate pathway, partially reversed statin-induced inhibition of YT-INDY proliferation and cytotoxicity. These results suggest that blockage of products made in the latter part of the mevalonate pathway may account for the observed inhibitory effects on YT-INDY proliferation and cytotoxicity. However, geranylgeranyl pyrophosphate could not reverse the statin-induced inhibition of the cell cycle.

**Conclusions:**

These results suggest that the statin drugs should be investigated as a potential therapeutic strategy for human natural killer cell leukemias possibly in combination with chemotherapeutic agents.

## Background

Human natural killer cells are a population of large granular lymphocytes which can lyse tumor target cells *in vitro* without prior sensitization and with no major histocompatibility complex restriction [1]. Lymphoproliferative diseases of large granular lymphocytes can arise from T lymphocytes or natural killer cells. Large granular lymphocyte leukemias derived from T lymphocytes generally follow an indolent course with a median survival time exceeding 10 years. However, large granular leukemias involving natural killer cells are typically very aggressive and patients have a median survival time of two months due to being refractory to multi-agent chemotherapy [2]. Most aggressive natural killer cell leukemias are associated with Epstein-Barr virus, but Epstein-Barr virus-negative natural killer cell leukemias, while rare, follow an indolent course. The World Health Organization classifies natural killer cell malignancies into three categories, including aggressive natural killer cell leukemia, extranodal natural killer lymphoma and blastic natural killer cell lymphoma [3].

Though studies are sparse, natural killer cell malignancies seem to be relatively rare in Caucasians, but the incidence rises significantly in Asian and South American populations [4,5]. One study from China showed that natural killer cell malignancies may account for around 6% of the natural killer and T cell malignancies [6].

Chemotherapy of aggressive natural killer cell leukemias tends to be ineffective owing to the expression of tumor cell P-glycoprotein, an ATP-dependent drug efflux pump. Based on the typical poor prognosis following chemotherapy, stem cell therapy is the treatment of choice for advanced disease [5]. Because efficacious therapy is not currently available for natural killer cell malignancies, it is imperative that new treatment strategies be investigated.

One potentially promising approach is through the use of statin drugs. Statins have been used to lower LDL cholesterol levels in those with hypercholesterolemia and have been shown to have a limited number of side effects. Their mechanism of action relies on interrupting the formation of cholesterol in the mevalonate pathway by inhibiting HMG-CoA reductase, which is responsible for controlling the rate of cholesterol production in the liver [7]. In addition, statins increase the ability of the liver to remove LDL-cholesterol that is present in the blood. It is not unusual for the LDL-cholesterol levels to drop by 20-60% in statin-treated patients. Commonly prescribed statins include lovastatin, simvastatin, atorvastatin, fluvastatin, pravastatin, and rosuvastatin calcium.

Recently, statins have been observed to have potentially beneficial effects beyond their ability to lower cholesterol levels. Studies have show that statins can have anti-inflammatory and immunomodulatory effects [8-10]. In addition, statins may have potent anti-tumor properties [11,12]. Investigations using tumor cell lines noted that statins inhibited the growth of acute myeloid leukemia cells [13], induced apoptosis in IM-9 human lymphoblasts [14], and increased the killing of the human erythroleukemia cell line K562 by a commonly used chemotherapeutic agent [15].

In terms of the effect of statins on natural killer cell leukemias, a clinical case report noted that treatment with a farnesyltransferase inhibitor, which interrupts the mevalonate pathway farther downstream than the statins, resulted in clinical improvement and, importantly, a reduction of pulmonary artery hypertension [16]. The pulmonary hypertension has been connected to damage to the pulmonary endothelial cells as a result of leukemic natural killer cell cytotoxicity. The novel effect of statins on leukemia cells is a new and potentially exciting field of research.

Our laboratory utilized the cell line YT-INDY to investigate the effect of statins on natural killer leukemic cells. The parental YT cell line is an interleukin-2-independent human leukemia natural killer cell line that has been used in numerous studies to investigate normal natural killer cell function. YT cells were isolated originally from a 15 year old boy with acute lymphoblastic lymphoma [17]. The cell line exhibits several natural killer cell surface markers, but does not express T lymphocyte markers such as CD3, CD4 or CD8. The T cell antigen receptor genes are in the germline configuration [18], which confirms that these cells are not T lymphocytes. Our laboratory used a cloned derivative of the YT cell line, named YT-INDY.

We performed these investigations because aggressive natural killer cell leukemias are devastating diseases which are nearly always fatal within weeks or, at best, a few months of diagnosis. Multi-agent chemotherapy has failed in the majority of patients with this disease. Therefore, new approaches to treatment must be developed that can provide a cure or significant life extension.

The YT-INDY natural killer leukemia cell line is an excellent pre-clinical model for studying the mechanisms behind statin-mediated inhibition of tumor cell functions. These functions include natural killer cytotoxicity, cell proliferation and cell cycle progression. We have demonstrated that several statin drugs can adversely impact the aforementioned cell functions of YT-INDY.

## Methods

### Chemicals

The following reagents were used in these studies: mevalonate (purchased as mevalonolactone from Sigma-Aldrich Co. LLC), geranylgeranyl pyrophosphate (Sigma-Aldrich Co. LLC), dimethylallyl pyrophosphate (Sigma-Aldrich Co. LLC), farnesyl pyrophosphate (Sigma-Aldrich Co. LLC), isopentenyl pyrophosphate (Sigma-Aldrich Co. LLC), squalene (Sigma-Aldrich Co. LLC), atorvastatin (Toronto Research Chemicals Inc.), fluvastatin (Selleck Chemicals Inc.), mevastatin (CalBiochem), simvastatin (CalBiochem), lovastatin (CalBiochem).

### Cell proliferation assay

Briefly, 1 × 10^5^ YT-INDY cells/ml were cultured in 25 cm^2^ culture flasks under sterile conditions in 5 ml of RPMI-1640 medium (Mediatech, Inc.). A single addition of statin drug was added to duplicate flasks at the start of the experiment at four concentrations, including 50 µM, 25 µM, 12.5 µM, and 6.2 µM. YT-INDY treated with the vehicle in which each statin was dissolved, served as controls. Cell counts were performed on each flask at 24 hour intervals for 72 hours total. Results were calculated as the percent inhibition compared to the untreated control. Each experiment was performed at least four times and the data was analyzed for statistical significance by Student’s t-test.

### Reversal of statin-mediated inhibition of proliferation

1 × 10^5^ YT-INDY cells/ml were cultured in 25 cm^2^ culture flasks under sterile conditions in 5 ml of RPMI-1640 medium. A single addition of simvastatin (50 µM) or fluvastatin (50 µM) was added to duplicate flasks at the start of the experiment. YT-INDY treated with the vehicle in which each statin was dissolved, served as controls. Some statin-treated flasks also received mevalonate (1 mM) or geranylgeranyl pyrophosphate (10 µM). Cell counts were performed on each flask at 24 hour intervals for 72 hours total. Each experiment was performed three times and the data was analyzed for statistical significance by Student’s t-test.

### Natural killer cell cytotoxicity assay

1 × 10^5^ YT-INDY cells/ml were cultured in 25 cm^2^ culture flasks under sterile conditions in 5 ml of RPMI-1640 medium. A single addition of statin drug was added in duplicate flasks at the start of the experiment at 25 µM, 12.5 µM, 6.2 µM or 3.1 µM concentrations. YT-INDY treated with the vehicle in which each statin was dissolved, served as controls. After a 24 hr. incubation, the cells were harvested and washed three times in Hank’s balance salt solution (Mediatech, Inc.) and resuspended in calf serum-supplemented RPMI-1640 medium at a concentration of 4 × 10^6^ cells/ml for use in the natural killer cell cytotoxicity assay. **N**atural killer cell cytotoxicity assays were performed as follows according to the technique of Steele and Brahmi [19,20]. Briefly, the YT-INDY-sensitive tumor cell line Phebo was used as the target cell in all cytotoxicity assays. 6 × 10^6^ Phebo, suspended in 0.5 ml RPMI-1640 medium, was incubated for 60 minutes at 37°C in the presence of 200 uCi sodium chromate (^51^chromium; Perkin-Elmer Life Sciences). The target cells were washed three times in Hank’s balance salt solution and resuspended at 1 × 10^5^ cells/ml in calf serum-supplemented RPMI-1640. Radiolabeled Phebo (100 µl) was added to each well of a 96-well microtiter plate, followed by 100 µl of YT-INDY (4 × 10^6^/ml) to yield a 40:1 YT-INDY to Phebo ratio. The plates were incubated for four hours in a 37°C, 5% CO_2_, humidified atmosphere. The plates were then centrifuged at 150 × g for 2 minutes and the supernatant from each well harvested using a Supernatant Collection System (Skatron, Inc.). The percent lysis of the Phebo target cells was computed as follows:

$$\%\mathrm{lysis}=\;\frac{\mathrm{test}\;\mathrm{cpm}‒\mathrm{spontaneous}\;\mathrm{cpm}\;}{\mathrm{maximum}\;\mathrm{cpm}‒\mathrm{spontaneous}\;\mathrm{cpm}}\;\times \;100$$

The maximum ^51^chromium contained in the target cells was determined by measuring the radioactivity in 75 µl radiolabeled Phebo. Measurement of radioactivity in the supernatant of wells containing only labeled target cells, following the 4 hour assay, yielded the spontaneous release. Percent inhibition of cytotoxicity was derived by comparing the inhibited value to the uninhibited control value. Statistical analysis was performed using Student’s t-test.

### Reversal of statin-mediated Inhibition of Cytotoxicity

The cytotoxicity assay, as described above, was utilized to determine whether statin-mediated inhibition of cytotoxicity could be reversed by intermediates of the mevalonate pathway. YT-INDY was incubated with 12.5 µM lovastatin in the presence or absence of mevalonate (1 mM or 0.5 mM), geranylgeranyl pyrophosphate (10 µM), dimethylallyl pyrophosphate (10 µM), farnesyl pyrophosphate (10 µM), isopentenyl pyrophosphate (10 µM) or squalene (200 µM) for 24 hours. YT-INDY treated with the vehicle in which each statin was dissolved, served as controls. Each experiment was performed three times and the data was analyzed for statistical significance by Student’s t-test.

### Cell cycle analysis

1 × 10^6^ YT-INDY, that had been incubated with or without statin drug for 72 hours, were washed three times in Hank’s balanced salt solution and labeled with propidium iodide (Sigma-Aldrich Inc. LLC). The cells were analyzed by flow cytometry using a Becton-Dickison FACScan instrument. Quantitation of the percentage of cells in the G0-G1, S and G2-M phases of the cell cycle was accomplished using the ModFit™ software package.

## Results

### Statins inhibit YT-INDY proliferation

**Figure 1:**
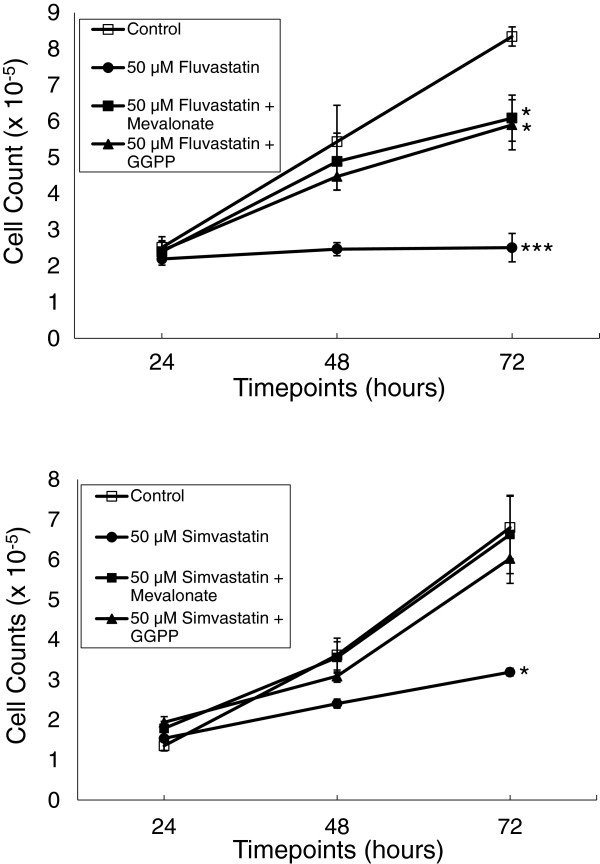
**Mevalonate and geranylgeranyl pyrophosphate reversed fluvastatin- and simvastatin-mediated inhibition of proliferation.** YT-INDY was incubated for up to 72 hours in the presence of fluvastatin (50 µM), simvastatin (50 µM) or the solvents in which the drugs were dissolved. The statins or vehicles were added at the start of the experiment. Some statin-treated flasks also received mevalonate (1 mM) or geranylgeranyl pyrophosphate (GGPP) (10 µM). Cell counts were performed on each flask at 24 hour intervals. Each experiment was performed three times and the data was analyzed for statistical significance by Student’s t-test. Statistical significance is denoted by asterisks. *: p < 0.05, ***: p < 0.001.

**Figure 2:**
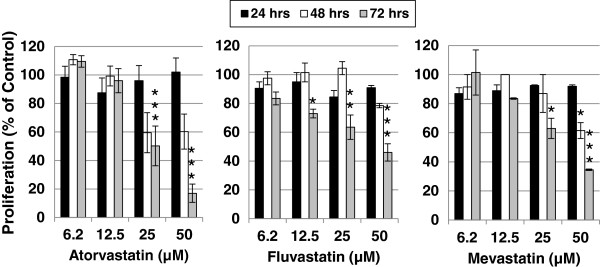
**Time-dependent inhibition of YT-INDY proliferation by statins.** YT-INDY was incubated for up to 72 hours in the presence of various concentrations atorvastatin, fluvastatin, or mevastatin. Control cells were treated with the solvents in which the drugs were dissolved. The statins or solvent were added at the start of the experiment. Cell counts were performed at 24 hour intervals. Results were calculated as the percent inhibition compared to the untreated control. Each experiment was performed at least four times and the data was analyzed for statistical significance by Student’s t-test. Statistical significance is denoted by asterisks. *: p < 0.05, **: p < 0.01, ***: p < 0.001.

To determine the effect of statins on the proliferation of YT-INDY, we incubated the cell line with 50 µM to 6.2 µM atorvastatin, fluvastatin, mevastatin, simvastatin (50 µM only; see Figure 1), lovastatin (data not shown) or the vehicle in which each was diluted and quantitated the growth of YT-INDY daily over a 72 hour period (Figure 2). Compared to controls, each of the statins exhibited a dose-related effect on the growth of the cell line. Though an inhibitory effect was observed as early as 48 hours, the most pronounced inhibition occurred at 72 hours. In order to confirm that the statins were acting through the mevalonate pathway, we were able to partially reverse the fluvastatin- or simvastatin-mediated inhibition of YT-INDY growth by the addition of 1 mM mevalonate or 10 uM geranylgeranyl pyrophosphate (Figure 1). Mevalonate overrides the statin inhibition early in the pathway, whereas geranylgeranyl pyrophosphate occurs late in the pathway.

### Statins impede cell cycle progression of YT-INDY

Given that statins inhibited YT-INDY cell proliferation, it seemed likely that we would observe statin-induced effects on YT-INDY cell cycle progression. Table 1 illustrates the effect on cell cycle progression when YT-INDY was incubated for 72 hours with 50 µM simvastatin, fluvastatin or lovastatin followed by cell cycle analysis by flow cytometry. Compared to controls, the statins increased the percentage of cells that were in the G0/G1 phase of the cell cycle, while decreasing the S phase. With the exception of fluvastatin, the other statins produced a modest increase in the G2/M phase. These effects could be completely reversed by the addition of mevalonate. Unlike its ability to reverse the statin-induced inhibition of cell proliferation, geranylgeranyl pyrophosphate had only a slight ability to reverse the inhibitory effect of the statins on the cell cycle.

**Table 1:** Statins can inhibit cell cycle progression and mevalonate can reverse the inhibitory effect

** Statins Reduce YT-INDY Cell cycle progression**			
	**Cell cycle phases**		
	G0/G1 (%)	G2/M (%)	S (%)
Control	36.1 ± 0.44^a^	10.6 ± 0.53	53.3 ± 0.69
50 µM Simvastatin	**49.1 ± 0.30**^**b**^	**13.1 ± 1.01**	**37.8 ± 0.97**
50 µM Simvastatin + Mevalonate	**39.9 ± 0.36**	**13.8 ± 0.48**	**46.3 ± 0.66**
50 µM Simvastatin + GGPP	**47.9 ± 0.64**	13.6 ± 0.32	**38.5 ± 0.87**
Control	29.3 ± 0.80	6.8 ± 0.21	63.9 ± 0.97
50 µM Fluvastatin	**41.7 ± 2.02**	**21.4 ± 1.67**	**36.9 ± 0.77**
50 µM Fluvastatin + Mevalonate	33.9 ± 1.60	6.7 ± 0.15	59.4 ± 1.62
50 µM Fluvastatin + GGPP	**42.1 ± 2.08**	10.4 ± 0.43	**47.5 ± 1.24**
Control	27.9 ± 1.00	5.6 ± 1.03	66.5 ± 0.15
50 µM Lovastatin	**50.4 ± 0.62**	**10.7 ± 0.84**	**38.9 ± 0.28**
50 µM Lovastatin + Mevalonate	30.2 ± 0.15	3.7 ± 0.32	66.1 ± 0.47
50 µM Lovastatin + GGPP	**39.6 ± 0.90**	8.2 ± 0.72	**52.2 ± 0.92**

^a^Values represent the means ± S.E.M. from three separate experiments.

^b^Bolded values represent statistical significance at least p < 0.05 compared to control values.

YT-INDY, incubated with or without statin drug or statin solvent, and with or without mevalonate or geranylgeranyl pyrophosphate (GGPP), were subjected to cell cycle analysis by flow cytometry.

### Statins inhibit YT-INDY cytotoxicity

**Figure 3:**
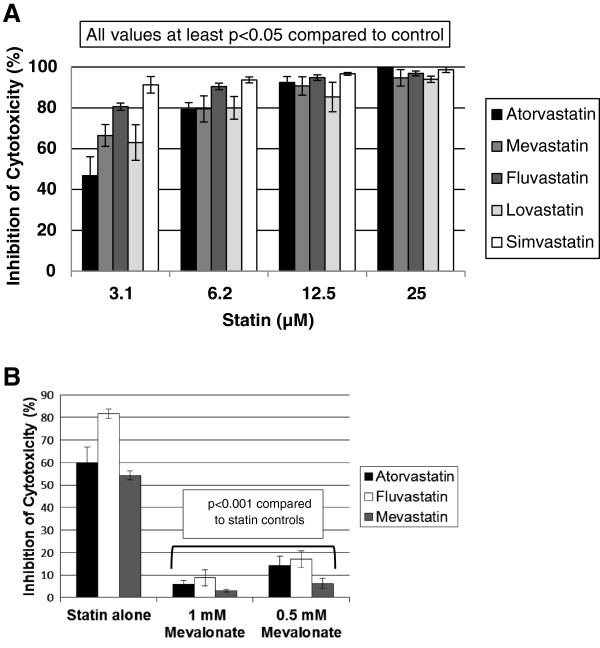
**Statins abrogate YT-INDY cytotoxicity against a tumor target cell line in a cytotoxicity assay.** A single addition of statin (25 µM, 12.5 uM, 6.2 µM or 3.1 µM), or solvent in which the statins were dissolved, was added to YT-INDY cells at the start of the experiment. After a 24 hour incubation period, the cells were harvested, washed and utilized in the 4 hour cytotoxicity assay. Some statin-treated cells (3.1 µM for each statin) also included the addition of mevalonate (1 mM or 0.5 mM). Statistical analysis was performed using Student's t-test. *Panel****A*** demonstrates the inhibition of YT-INDY cytotoxicity compared to the control. All values shown are statistically significant. *Panel****B*** shows the reversal of statin-mediated inhibition of cytotoxicity in the presence of mevalonate. All mevalonate values were highly statistically significant.

Because natural killer cytotoxicity may play a role in the pulmonary hypertension seen in some natural killer leukemia patients, we were interested in determining the effect of statins on YT-INDY cytotoxicity. Five statins, ranging from 25 µM to 3.1 uM, profoundly inhibited YT-INDY cytotoxicity at all concentrations tested (Figure 3A). 1 mM mevalonate was found to be the best concentration to full reverse the effects of the atorvastatin, fluvastatin and mevastatin treatments, with each statin being used at a concentration of 3.1 µM (Figure 3B). This demonstrated that the effects of statins were mediated through the mevalonate pathway.

### Reversal of statin-mediated inhibition of cytotoxicity using mevalonate pathway intermediates

**Figure 4:**
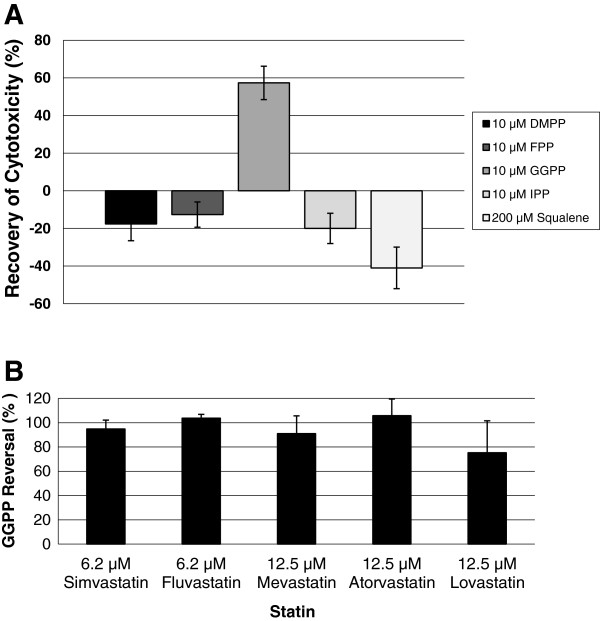
**Reversal of statin-mediated inhibition of cytotoxicity by geranylgeranyl pyrophosphate.** The natural killer cell cytotoxicity assay was used to determine whether statin-mediated inhibition of cytotoxicity could be reversed by intermediates of the mevalonate pathway. YT-INDY was incubated with lovastatin (12.5 µM) in the presence or absence of geranylgeranyl pyrophosphate (GGPP) (10 µM), dimethylallyl pyrophosphate (DMPP) (10 µM), farnesyl pyrophosphate (FPP) (10 µM), isopentenyl pyrophosphate (IPP) (10 µM) or squalene (200 µM) for 24 hours. YT-INDY treated with the solvent in which each statin was dissolved, served as controls. Each experiment was performed three times and the data was analyzed for statistical significance by Student’s t-test. *Panel****A*** demonstrates that only geranylgeranyl pyrophosphate could reverse the lovastatin-mediated inhibition of cytotoxicity. *Panel****B*** indicates that geranylgeranyl pyrophosphate could reverse the abrogation of cytotoxicity caused by five different statins.

In order to investigate which mevalonate pathway intermediate was most effective in reversing the inhibition of YT-INDY cytotoxicity, various intermediates were used in combination with lovastatin and compared to lovastatin alone controls. Mevalonate pathway intermediates included 10 µM concentrations of geranylgeranyl pyrophosphate, dimethylallyl pyrophosphate, farnesyl pyrophosphate or isopentenyl pyrophosphate. In addition we included squalene as a molecule important in the production of cholesterol. We demonstrated geranylgeranyl pyrophosphate alone was effective in reversing the inhibition of YT-INDY cytotoxicity by lovastatin (Figure 4A). We then tested five statins with geranylgeranyl pyrophosphate to determine whether it would reverse all statin-induced inhibition of YT-INDY cytotoxicity. Figure 4B shows that geranylgeranyl pyrophosphate had the capability to significantly reverse all statin-mediated inhibition.

## Discussion

In these investigations, we found that various statin drugs could inhibit YT-INDY proliferation, cell cycle progression and cytotoxicity. These novel effects of statins may be of clinical importance given that natural killer cell leukemias tend to be very difficult to treat with standard chemotherapy regimens. The only approach that has been shown to be successful with some aggressive natural killer cell leukemias has been allogeneic bone marrow transplantation [3,21]. Given that life spans following the diagnosis of an aggressive natural killer cell leukemia is generally measured in months [2], patients need more effective approaches for this type of leukemia.

We observed a dose-related effect of the statins on the proliferation of YT-INDY that started to become apparent within 48 hours of administration and was highly significant at the higher concentration of statins within 72 hours. This rapid response to statins was encouraging since the statins were only administered at the start of the experiment. It is important to note that, unlike the other statins tested, mevastatin is not used in humans due to its toxic side effects. Reversal of the inhibition by mevalonate or geranylgeranyl pyrophosphate signified that the statins exerted their effect through inhibition of HMG CoA reductase.

Based on the inhibition of proliferation, we investigated the effect of statins on the cell cycle of YT-INDY because it stood to reason that it might be adversely impacted by statin treatment. Our results indicated that simvastatin, fluvastatin and lovastatin profoundly altered the number of cells in the G0/G1 and S phases of the cell cycle. The increase in the G0/G1 phase and the decrease in the S phase is consistent with the finding of decreased cellular proliferation. Interestingly, mevalonate could restore normal cell cycle function, but geranylgeranyl pyrophosphate was unable to do so. This is direct contrast to the observation that both compounds can reverse statin-mediated inhibition of proliferation. The significance of this finding is not currently known.

Because YT-INDY is derived from the natural killer cell lineage, the cell line exhibits significant cytotoxicity against some tumor target cells. Unfortunately, one adverse effect of these leukemias is the cytotoxicity-mediated destruction of the vascular endothelial cells of the respiratory tract leading to the development of pulmonary hypertension [16]. Therefore, we were interested in determined whether statins could diminish the cytotoxicity of YT-INDY cells. Experiments revealed that YT-INDY cytotoxicity was exceptionally sensitive to inhibition by the statins. Atorvastatin, mevastatin, fluvastatin, lovastatin and simvastatin was able to inhibit much of the cytotoxic activity of YT-INDY even at clinically significant concentrations of statin. Mevalonate, at 1 mM concentration, was able to fully restore YT-INDY cytotoxicity, indicating that the statin-mediated inhibition occurred through the mevalonate pathway. Because statins can significantly decrease natural killer cell leukemia cytotoxicity, statin therapy might be of benefit to patients, particularly in terms of potentially decreasing the damage to the pulmonary vascular endothelial cells.

To more fully understand the part of the mevalonate pathway that might be affected by statins to alter the cytotoxic activity of YT-INDY, we investigated whether adding back various intermediates of the pathway would decrease the statin-induced inhibition of cytotoxicity. We chose lovastatin as the statin as it exhibited a medium level of cytotoxicity inhibition at 3.1 µM, compared to the other statins. Of all the intermediates tested, it is noteworthy that only geranylgeranyl pyrophosphate was able to reverse the inhibition of cytotoxicity induced by lovastatin. The majority of statin-mediated inhibition of YT-INDY could be restored by geranylgeranyl pyrophosphate when tested against atorvastatin, mevastatin, fluvastatin, lovastatin and simvastatin. Squalene, a precursor of cholesterol, couldn’t reverse the statin-induced inhibition, suggesting that cholesterol was not a necessary component in the cytotoxic process. While it is not known why other mevalonate pathway intermediates could not reverse the effects of the statins, it may be that the stage at which a particular intermediate feeds into the pathway might allow the siphoning off of that intermediate into other products prior to the production of products that are needed for the cytotoxic process.

In summary, our results suggest that statins should be investigated as a potential co-therapy, along with chemotherapeutic agents, for natural killer cell leukemias. One significant advantage of testing statins in natural killer cell leukemia patients is that the drugs are already approved for use in patients to treat hypercholesterolemia and the toxicity profile and side effects are well known. Studies are currently underway to investigate whether the combination of statins and chemotherapy is more effective than either compound alone.

## Conclusions

Aggressive natural killer cell leukemias are very difficult to treat due to their intrinsic resistance to chemotherapeutic agents, therefore new treatment strategies must be developed. Our investigations showed that statin drugs can inhibit the growth, cell cycle progression and cytotoxicity of the YT-INDY natural killer cell leukemia cell line. Based on these results, statin drugs should be investigated as a potential therapeutic strategy for human natural killer cell leukemias, possibly in combination with chemotherapeutic agents.

## Abbreviations

GGPP: Geranylgeranyl pyrophosphate; DMPP: Dimethylallyl pyrophosphate; FPP: Farnesyl pyrophosphate; IPP: Isopentenyl pyrophosphate.

## Competing interests

The authors declare that they have no competing interests.

## Authors’ contributions

TAS conceived of the study, participated in its design and coordination, secured funding and drafted the manuscript. JC and MM carried out the cytotoxicity and proliferation studies. RD and AI conducted the cell cycle analysis investigations and the reversal of statin-mediated inhibition of proliferation and cytotoxicity. All authors were involved in the statistical analysis of the results. All authors read and approved the final manuscript.

## Authors’ information

JC, MM, RD and AI were summer research students in the laboratory of TAS.
